# Identification of a peptide ligand for human ALDH3A1 through peptide phage display: Prediction and characterization of protein interaction sites and inhibition of ALDH3A1 enzymatic activity

**DOI:** 10.3389/fmolb.2023.1161111

**Published:** 2023-03-20

**Authors:** Georgia-Persephoni Voulgaridou, Vasileios Theologidis, Vasileios Xanthis, Eleni Papagiannaki, Ilias Tsochantaridis, Vasiliki E. Fadouloglou, Aglaia Pappa

**Affiliations:** Department of Molecular Biology and Genetics, Faculty of Health Sciences, Democritus University of Thrace, Alexandroupolis, Greece

**Keywords:** aldehyde dehydrogenase, ALDH3A1, phage display, protein-protein interactions, enzyme inhibitor

## Abstract

Aldehyde dehydrogenase 3A1 (ALDH3A1) by oxidizing medium chain aldehydes to their corresponding carboxylic acids, is involved in the detoxification of toxic byproducts and is considered to play an important role in antioxidant cellular defense. ALDH3A1 has been implicated in various other functions such as cell proliferation, cell cycle regulation, and DNA damage response. Recently, it has been identified as a putative biomarker of prostate, gastric, and lung cancer stem cell phenotype. Although ALDH3A1 has multifaceted functions in both normal and cancer homeostasis, its modes of action are currently unknown. To this end, we utilized a random 12-mer peptide phage display library to identify efficiently human ALDH3A1-interacting peptides. One prevailing peptide (P1) was systematically demonstrated to interact with the protein of interest, which was further validated *in vitro* by peptide ELISA. Bioinformatic analysis indicated two putative P1 binding sites on the protein surface implying biomedical potential and potent inhibitory activity of the P1 peptide on hALDH3A1 activity was demonstrated by enzymatic studies. Furthermore, in search of potential hALDH3A1 interacting players, a BLASTp search demonstrated that no protein in the database includes the full-length amino acid sequence of P1, but identified a list of proteins containing parts of the P1 sequence, which may prove potential hALDH3A1 interacting partners. Among them, Protein Kinase C Binding Protein 1 and General Transcription Factor II-I are candidates of high interest due to their cellular localization and function. To conclude, this study identifies a novel peptide with potential biomedical applications and further suggests a list of protein candidates be explored as possible hALDH3A1-interacting partners in future studies.

## 1 Introduction

Aldehyde dehydrogenase 3A1 (ALDH3A1) is a detoxifying cytoplasmic enzyme responsible for the oxidation of medium-chain aliphatic and aromatic aldehydes to their corresponding acids as well as for the metabolism of the chemotherapeutic agent cyclophosphamide (CP) (Jester, 2008; Parajuli et al., 2014a). ALDH3A1 is expressed in the liver as a response to certain xenobiotics while it is constitutively expressed in tissues that are regularly exposed to environmental stress, e.g., lung, stomach, bladder, skin, and cornea (Pappa et al., 2003). Its ability to efficiently detoxify a panel of toxic aldehydes, by-products of lipid peroxidation, is crucial for cellular antioxidant defenses.

Apart from its metabolic role though, several lines of evidence suggest that ALDH3A1 is a multifaceted protein with variable non-catalytic properties in both normal and cancer homeostasis. In the cornea, ALDH3A1 is expressed in a taxon-specific manner in most mammals. Due to its abundance (up to 50% of the water-soluble proteins in certain species), ALDH3A1 has been characterized as “corneal crystallin” and was proposed to contribute to the transparency and refraction properties of the tissue (Vasiliou et al., 2013; Voulgaridou et al., 2017). The idea that ALDH3A1 is a structural component of the cornea has been proved invalid by experimental data showing that ALDH3A1 knockout mice have corneas that appear identical to those of wild-type animals (Nees et al., 2002). Nevertheless, ALDH3A1 has a pivotal role in the maintenance of the tissue’s optical properties considering that Aldh3a1 knockout mice were susceptible to corneal edema and clouding both under normal and ultraviolet B (UVB) exposure conditions (Lassen et al., 2007; Chen et al., 2017). ALDH3A1 ability to protect the cornea from opacification implies that it would prevent the accumulation and/or precipitation of denatured/insoluble proteins under certain conditions. Indeed, previous studies indicated that ALDH3A1 exhibits a chaperone-like activity as it managed to protect the enzymes SmaI and citrate synthase from inactivation and/or precipitation under thermal stress conditions (Voulgaridou et al., 2017).

Additionally, certain findings suggest that ALDH3A1 is associated with cellular differentiation and cell cycle regulation. In the cornea, ALDH3A1 is expressed in the epithelial cells and the keratocytes of the stroma, both of which are post-mitotic, differentiated cells incapable of cellular division. In contrast, ALDH3A1 is absent from the undifferentiated stem and transient amplifying cells of the corneal endothelium (Pappa et al., 2003). Interestingly, in explant primary cultures of human corneal epithelium, ALDH3A1 expression was downregulated as a result of corneal epithelial cell proliferation (Pappa et al., 2005). Similarly, overexpression of ALDH3A1 in the human corneal epithelial cell line HCE resulted in slower proliferation rates, an elongated cell cycle as well as an imbalance of a panel of cell cycle regulators (Pappa et al., 2005). Novel experimental data by our group suggest that the DNA damage response signaling pathway (DDR) could be involved in the association of ALDH3A1 and cell cycle progression. Specifically, it was demonstrated that ALDH3A1 prevented the hydrogen peroxide (H_2_O_2_)-, tert-butyl peroxide- and etoposide-induced formation of DNA lesions in the human corneal epithelial cells HCE-2 (Voulgaridou et al., 2020). This protective effect was accompanied by an altered expression profile of a set of genes associated with DNA damage repair, apoptosis, and ATM/ATR signaling cascade (e.g., TP53, PRKDC, DDB1, H2AFX, and CDKN1A) (Voulgaridou et al., 2020). Finally, in addition to its cytoplasmic localization, ALDH3A1 has also been found at the nucleus of both ALDH3A1 transfected rabbit corneal keratocytes as well as human transfected epithelial cells (Pappa et al., 2005; Stagos et al., 2010). The nuclear localization of ALDH3A1 is of crucial importance as it implies a potential direct association of the protein in mechanisms of gene regulation.

Finally, ALDH3A1 has been characterized as a potential cancer stem cell (CSC) marker in various types of solid tumor malignancies. CSCs are cancer cell subpopulations possessing a range of stem-related properties such as multipotency, self-renewal, enhanced tumorgenicity, and increased radio/chemoresistance (Huang et al., 2009; Zhao, 2016; Schulz et al., 2019). Specifically, ALDH3A1 was found to be associated with CSCs in melanoma as well as in lung, prostate, and gastric cancer (Yan et al., 2014; Wu et al., 2016; Terzuoli et al., 2019). Considering the aggressiveness and the resistant phenotype of these cells, ALDH3A1 has emerged as a valuable marker in cancer research.

ALDH3A1 is a homodimer of two 453-long protein chains. Each chain folds into a catalytic and a cofactor binding domain. Both domains adopt a Rossmann fold. The substrate and cofactor binding sites are located in a tunnel which is mainly formed on the interface of the two domains. One entry of the tunnel is the cofactor binding site entry and the other is the substrate binding site entry. The substrate and cofactor sites are delimited at the interior of the tunnel by the catalytic residues Cys243 and Glu209. Cys243 attacks the substrate’s carbonyl group, and Glu209 participates in the hydrolyzation of the intermediate acyl-enzyme by a water molecule (Khanna et al., 2011). ALDH3A1 can bind either nicotinamide adenine dinucleotide (NAD^+^) or nicotinamide adenine dinucleotide phosphate (NADP^+^), in contrast to the majority of ALDH superfamily members that bind selectively only one of these dinucleotides (Shortall et al., 2021).

Due to the multifunctionality of ALDH3A1 in a wide spectrum of biological activities and its great clinical importance, we were prompted to work towards two research directions: a) to identify peptide (s) that interact with human ALDH3A1 (hALDH3A1) and thus could be further exploited for biomedical applications, and b) to utilize the identified peptide (s) for discovering putative protein interaction partners of hALDH3A1.

## 2 Materials and methods

### 2.1 Materials

Media for bacterial cultures along with antibiotics were purchased either from Applichem (Darmstadt, Germany) or Sigma-Aldrich Co. (Taufkirchen, Germany). Phage Display peptide library kit Ph.D.-12 was purchased from New England Biolabs (Beverly, MA, United States). Isopropyl β-d-1-thiogalactopyranoside (IPTG), 5-Bromo-4-chloro-3-indolyl β-D-galactopyranoside (X-gal), and DNA ladders were from Fermentas (Burlington, ON, Canada). The chemicals used for the phage display assay were either obtained from Sigma-Aldrich Co. (Taufkirchen, Germany), Applichem (Darmstadt, Germany), or Carl Roth GmbH (Karlsruhe, Germany). The chemicals used for the peptide Enzyme-linked Immunosorbent Assay (ELISA) were either obtained from Sigma-Aldrich Co. (Taufkirchen, Germany), Applichem (Darmstadt, Germany), or Carl Roth GmbH (Karlsruhe, Germany). The rabbit polyclonal antibody against human ALDH3A1 used in the experiment was purchased from Abnova (Taipei City, Taiwan), and the goat anti-rabbit IgG horseradish peroxidase-conjugated antibody was purchased from Millipore (Bedford, MA, United States). The reagents for the ALDH3A1 enzymatic activity were obtained by Sigma-Aldrich Co. (Taufkirchen, Germany). The synthetic peptide P1 was produced by Genecust (Boynes, France).

### 2.2 Phage Titration

For phage titration, 50 μL of an overnight *E. coli* ER2738 (host strain) culture were used for the inoculation of 5 mL Luria Broth (LB) which was subsequently cultivated at 37°C (220 rpm). When *E. coli* ER2738 culture reached an OD600 value of approximately 0.5, 200 μL of the culture were mixed with 10 μL of the phage solution and the mixture was vortexed and incubated for 5 min at room temperature (RT). Subsequently, it was diluted in 3 mL of warm agarose top (0.7% agarose, 0.5% yeast extract, 1% Bacto-Tryptone, 0.5% NaCl, 0.1% MgCl_2_•H_2_O) and poured in LB/IPTG/Xgal agar plates (LB agar plates with 0.004% X-gal 0.005% IPTG). Plates were incubated overnight at 37°C.

### 2.3 Isolation and sequencing of ssDNA from M13

The procedure was initiated with the amplification of the phage plaques as described in 2.5 with the exception that after the first centrifugation, 500 μL of the supernatant was transferred into a fresh tube. Then, 200 μL PEG/NaCl were added and the sample was mixed and incubated at RT for 10 min. The sample was subsequently centrifuged at 14,000 rpm, for 10 min at 4°C and the supernatant was removed. After the second round of centrifugation under the same conditions, the remaining supernatant was carefully discarded. Pellet was then re-suspended in 100 μL of iodine buffer (4M NaI, 1 mM EDTA, 10 mM Tris-HCl, pH 8.0) and 250 μL of ethanol were subsequently added. The mixture was incubated at RT for 10 min, centrifuged at 14,000 rpm for 10 min at RT, and the supernatant was discarded. Finally, 70% of pure ethanol was used for DNA wash and the pellet was suspended in 30 μL sterilized ddH_2_O. Samples were stored at −20°C until needed. Isolated M13 phage ssDNA (∼120 ng) was subjected to agarose gel electrophoresis, and the residual volume of the ssDNA was sent for single extension sequencing from the primer 5′-CCC​TCA​TAG​TTA​GCG​TAA​CG-3’. Sequencing was conducted by VBC biotech (Vienna, Austria). The resulting sequences were used for the estimation of the respective 12-mer peptides.

### 2.4 ALDH3A1 coating

For coating, human recombinant ALDH3A1, fused with 6 histidines in its carboxyl end, was expressed in its native form in *E. coli*, through chaperones’ co-expression (pG-KJE8 plasmid) and purified by affinity chromatography by using a nickel-nitrilotriacetic acid (Ni-NTA) column as described by Voulgaridou et al. (2013). The recombinant protein (75 μg) was diluted in 1.5 mL of coating buffer (0.1 M NaHCO_3_, pH 8.6), added to a Petri dish (60 mm), and incubated for 16 h at 4°C under gentle agitation. Following a 16 h incubation, the coating solution was removed; the plate was filled with blocking buffer (5 mg/mL bovine serum albumin (BSA) in 0.1 M NaHCO_3_, pH 8.6) and incubation was continued for 2 h at 4°C. The blocking solution was subsequently removed, and the plate was subjected to six wash cycles with TBST buffer (150 mM NaCl, 50 mM Tris-HCl, 1% Tween-20, pH 7.5).

### 2.5 Phage display (panning experiment)

**FIGURE 1:**
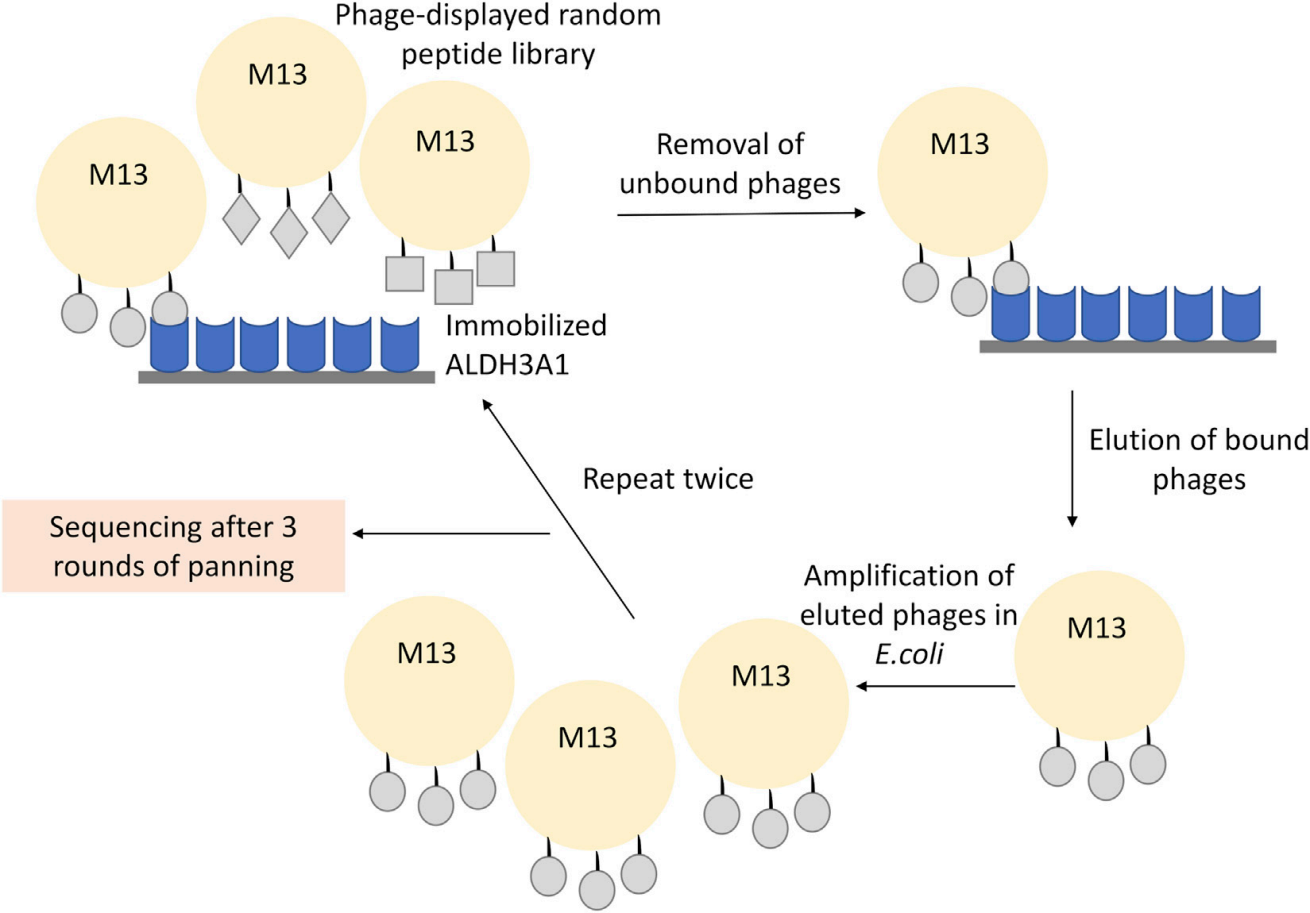
Representative scheme of the phage display experiment. The recombinant ALDH3A1 protein was immobilized at the surface of a plate and was subsequently incubated with the 12-mer peptide library displayed on the surface of the M13 phages. The unbound phages were removed through repetitive washes, while the bound phages were eluted under low pH conditions, amplified, and re-used into a new round of panning. To increase the specificity of the assay, three overall rounds of panning and amplification were conducted. Finally, the isolated phages from the last round were isolated and sequenced.

For phage display, 4 × 10^10^ phages of the 12-mer peptide phage display library were diluted in 1 mL TBST and plated in the ALDH3A1 coated dish for 60 min at RT under gentle shaking. The mixture of the non-binding phages was then removed, and the plate was washed 10 times with TBST. To elute the binding phages, the plate was incubated for 9 min with 1 mL of elution buffer (0.2 M Glycine-HCl; pH 2.2 containing 1 mg/mL BSA). The mixture was pipetted into a fresh microcentrifuge tube, neutralized with the addition of 150 μL of 1 M Tris-HCl, pH 9.1, and kept overnight at 4°C. Meanwhile, 5 mL of LB were inoculated with a single ER2738 colony and incubated O/N at 37°C, 220 rpm. The next day, 1 μL of the phage eluate was diluted in 10 μL of LB and used for phage titration (described in Phage Titration), while the rest of the phages in the eluate were amplified. For the amplification, the phages were added in 20 mL of LB inoculated with 200 μL of the overnight ER2738 culture. The mixture was incubated at 37°C, 220 rpm for 4.5 h, and then centrifuged at 10,000 rpm for 10 min at 4°C. The supernatant was subsequently transferred to a fresh tube and re-centrifuged once more under the same conditions. Then, 80% of the supernatant was transferred to a fresh tube and 1/6 of its volume of PEG/NaCl (20% polyethylene glycol-8000, 2.5 M NaCl) was added and incubated O/N at 4°C to precipitate phages. The mixture was then centrifuged at 10,000 rpm for 15 min at 4°C; the supernatant was removed and the sample was re-centrifuged under the same conditions. The residual supernatant was removed carefully and the precipitated phages in the pellet were resuspended in 1 mL TBS. The sample was subjected to another centrifugation at 14.000 rpm for 5 min at 4°C and 1/6 of the mixture’s volume of PEG/NaCl was used once more. The sample was incubated for 60 min at 4°C and then centrifuged at 14,000 rpm, for 10 min (4°C); the supernatant was removed and centrifuged under the same conditions. The supernatant was removed and the pellet was suspended in 200 μL TBS, 0.02% NaN3. Final centrifugation at 14,000 rpm for 1 min (4°C) was performed to assure the purity of the supernatant. The isolated phages were reused for another round of panning (Figure 1).

In the third round of panning, 1 μL of the eluted phages was diluted in serial dilutions from 1:10 to 1:10^7^ at a final volume of 10 μL LB broth and was used for phage tittering. The resulting LB/IPTG/Xgal agar plate was used for the amplification of the selected clones and the characterization of the peptides which showed interaction with ALDH3A1.

### 2.6 Phage amplification

Exactly 33 blue single plaques of phages were selected from the plate which was titered by the 1:10^7^ diluted final eluate. For amplifying the phages, 1 mL of LB broth was inoculated with 10 μL of an overnight *E. coli* ER2738 culture. Meanwhile, by using a sterile pipette tip, a well-partite blue plaque was stabbed and dropped into the culture tube which was subsequently incubated at 37°C, 220 rpm for 4.5 h. All cultures were then centrifuged at 14,000 rpm, for 30 s, the supernatant was removed, and samples were re-spun under the same conditions. Approximately 80% of the supernatant, which was the amplified phage, was transferred to a fresh tube and stored until needed at −20°C after adding glycerol at a dilution of 1:1.

### 2.7 Peptide ELISA

An ELISA with the synthetic peptide P1 was performed as described (Thermo Scientific, 2010). P1 (50 μg/mL) diluted in Coating Buffer (0.2 M sodium carbonate/bicarbonate, pH 9.4) was coated overnight at 4°C under agitation. After three washes with TBST (25 mM Tris, 0.15 M sodium chloride, pH 7.2 containing 0.1% Tween 20), the wells were blocked with 300 μL per well with Blocking Buffer (TBST containing 4% w/v BSA) overnight at 4°C under agitation. After another three washes with TBST, 100 μL of 2-fold serial dilutions of ALDH3A1 in Blocking Buffer were added in each well and the plate was incubated for 1 h at RT under gentle agitation. Following four washes with TBST, 100 μL of rabbit anti-ALDH3A1 antibody, diluted 1:1,000 in Blocking Buffer, were added and the plate was incubated for 2 h at RT under gentle agitation. After the plate was washed 4 times with TBST, it was incubated with 100 μL of anti-rabbit IgG horseradish-peroxidase conjugated and diluted 1:5,000 in Blocking Buffer, for 2 h at RT. Then, the plate was washed 6 times with TBST, and 100 μL of ABTS [2,2′-azino-bis(3-ethylbenzothiazoline-6-sulfonic acid)] solution (ready-to-use) were added. After 1 h of incubation in darkness at RT under agitation, the plate was measured at 405 nm, with wavelength correction at 650 nm, using an ELISA plate reader (EnSpire Multimode Plate Reader, Perkin Elmer, Waltham, MA, United States). During wash steps, liquids were dispersed in the sink, the plate was tapped vigorously on a paper towel, refilled with Wash Buffer, and incubated for 5 min at RT under agitation.

### 2.8 ALDH3A1 enzymatic assay

The ability of ALDH3A1 to oxidize benzaldehyde in the presence of P1 was estimated by monitoring the production of NADH as described earlier with minor modifications (Voulgaridou et al., 2013). Human recombinant ALDH3A1 was combined with P1 and diluted in Milli-Q water, at 1:1,000. The final reaction was placed in a 0.5 mL quartz cuvette containing 50 mM Tris-HCl pH 8.5, 50 mM 2-Mercaptoethanol, 1.5 mM NADH+, 20 nM ALDH3A1, 20 μM P1, and 1 mM Benzaldehyde. The reaction was initiated by the addition of the benzaldehyde substrate and was monitored at 340 nm for 5 min using a spectrophotometer (Libra S22, Biochrom Ltd, Cambridge, United Kingdom). In the case of CB29, the final reaction contained 40 μM CB29. Enzymatic activity was calculated as

$$dOD/\left(dT*molarextinctioncoeiffcient*pathcorrection*{enzyme}^{\prime }sconcentrationinfinalreaction\right)$$

where, dT = minutes, molar extinction coefficient = 6220 M^-1^/cm, path correction = 1 cm.

All experiments were conducted in triplicates and presented as % of the control’s enzymatic activity. As a control, the final reaction contained the respective Milli-Q water volume without P1 or CB29.

### 2.9 Modeling of protein-peptide interactions

Peptide docking calculations on the dimer of hALDH3A1 (PDB id 3SZA) were performed by the web servers HPEPDOCK (http://huanglab.phys.hust.edu.cn/hpepdock/) (Zhou et al., 2018) and CABS-dock (http://212.87.3.12/CABSdock/) (Kurcinski et al., 2015; Blaszczyk et al., 2016). The top 10 generated models (protein-peptide complex structures) from each algorithm were further evaluated by visual inspection and structural analysis. HPEPDOCK is based on a hierarchical algorithm, which functions by quickly sampling the different peptide conformations and docking them onto the target protein (Zhou et al., 2018). The predicted models are ranked according to their docking energy scores (see Table 2). CABS-dock performs protein-peptide docking by allowing full flexibility of the peptide on the entire protein surface without requiring information about the peptide structure or binding site (Kurcinski et al., 2015; Blaszczyk et al., 2016). The predicted models are ranked largely based on the outcome of structural clustering. CABS-dock estimates the docking quality and compares forecasting errors of the models by assessing the root mean square deviation (RMSD) value (Table 2). Lower RMSD values indicate better binding interactions and subsequently higher docking pose quality. The 3D structures were visualized with PyMOL (The PyMOL Molecular Graphics System, Schrödinger, LLC).

The top five models that dock P1 at Site 1 (1-4 from HPEPDOCK and 1 from CABS-dock) and the 2 models that dock P1 at Site 2 (5 from HPEPDOCK and 4 from CABS-dock) were analyzed by PROtein binDIng enerGY prediction (PRODIGY, https://wenmr.science.uu.nl/prodigy/) to identify the intermolecular contacts at the interface within the threshold distance of 5.5 Å (Vangone and Bonvin, 2015; Xue et al., 2016). GraphPad Prism software (version 8.3.0) was used for the construction of the heatmaps.

### 2.10 Multiple sequence alignment

The sequences of all the hALDH superfamily members were aligned using the Τ-Coffee algorithm (http://tcoffee.crg.cat/apps/tcoffee/do:regular) (Notredame et al., 2000). The results of the alignment were viewed on the Jalview software (Waterhouse et al., 2009) to identify the conserved regions within sequences.

### 2.11 A P1-based search of protein databases

The sequence of P1 was used in a BLASTp search against PDB, UniProt, RefSeq, and the non-redundant protein databases to find proteins that include this peptide in their sequences. The search was performed at https://blast.ncbi.nlm.nih.gov/Blast.cgi site (Camacho et al., 2009) of the National Center for Biotechnology Information (NCBI) and was limited by setting the “Organism” parameter to “*Homo sapiens*” (taxid: 9,606).

### 2.12 Protein-protein docking

The structures of Protein kinase C-binding protein 1 (PRKCBP1, PDB id 5B73) and the General Transcription Factor II-I (GTFII-I, PDB id 2ED2) were used in docking calculations with hALDH3A1 structure (PDB id 3SZA) in order possible protein-protein interaction interfaces to be predicted. For these calculations, the GalaxyTongDock_A (http://galaxy.seoklab.org/cgi-bin/submit.cgi?type=TONGDOCK_INTRO) was used, which is a tool for asymmetric docking of two different proteins (Park et al., 2019). The search was limited to the Z1 and G1 containing areas of PRKCBP1 and GTFII-I proteins, respectively. On the contrary, no restrictions were imposed on hALDH3A1. PISA at the European Bioinformatics Institute (PDBePISA) was used to further analyze and evaluate the predicted interfaces (http://www.ebi.ac.uk/pdbe/prot_int/pistart.html) (Krissinel and Henrick, 2007). In addition, the predicted protein-protein complexes with the higher docking scores were further analyzed by PRODIGY to determine their binding affinity (ΔG) and their dissociation constant (K_d_) at 37°C.

Motif Scan by MyHits (https://myhits.sib.swiss/cgi-bin/motif_scan) (Pagni et al., 2007) was used for scanning the hALDH3A1 sequence for all known motifs that can be recognition sites by other proteins, and for domain signatures through which it may interact with other proteins.

### 2.13 Statistical analysis

At least three independent experiments were performed for each condition tested. The values are expressed as the mean ± standard deviation (SD). Statistical analysis was performed with Graph Pad Prism software (version 9.4.1) (Graph Pad Inc., La Jolla, CA, United States). For all assays, statistical differences between groups were evaluated either by Student’s t-test or ANOVA, for multiple comparisons, followed by Dunnett’s or Tukey’s test. A level of *p* < 0.01 was considered statistically significant.

## 3 Results

### 3.1 Phage display led to one prevailing 12-mer peptide, WPTYVSPFRSPP, which appeared in 30 out of the 33 isolated clones

To examine the putative protein interactions of human ALDH3A1, we utilized the *in vitro* technique phage display and in particular the Phage Display peptide library kit Ph.D.-12 comprised of a library of 12-mer peptides fused to the N-final pIII coat protein of M13. The library harboring degenerate sequences (2.7 × 10^9^) was electroporated into host bacteria resulting in around five copies of its sequence per 1 μL of the supplemented phage library. For phage display, 75 μg of human recombinant ALDH3A1 were coated onto a sterile polystyrene Petri dish (100 mm) and 10 μL of the phage library were subsequently used for the first panning experiment. After three overall cycles of panning (Figure 1), the eluted pool of phages was serially diluted from 1:10 to 1:10^7^ and tittered onto LB/IPTG/Xgal plates. From the 1:10^7^ dilution plate, 33 blue single plaques of phages were selected and amplified and their ssDNA was isolated and sequenced. Sequencing results revealed four different peptides with one of them appearing in 30 out of 33 samples (Table 1). The WPTYVSPFRSPP peptide hereafter referred to as P1, appears to exhibit the strongest and most specific interaction with the recombinant hALDH3A1 due to its extremely high frequency of appearance. Therefore, the subsequent analysis was mainly focused on this peptide. The peptide P1 was further synthesized and tested for its potential interaction with recombinant hALDH3A1 protein.

**TABLE 1:** Phage display led to the identification of four (4) peptides.

Peptide	Frequency of appearance
WPTYVSPFRSPP	30:33
WPTSLTSAQFLF	1:33
ALHPLTNRHYAT	1:33
TPFPFAPLGRPP	1:33

### 3.2 Peptide ELISA confirmed the binding of P1 to hALDH3A1

**FIGURE 2:**
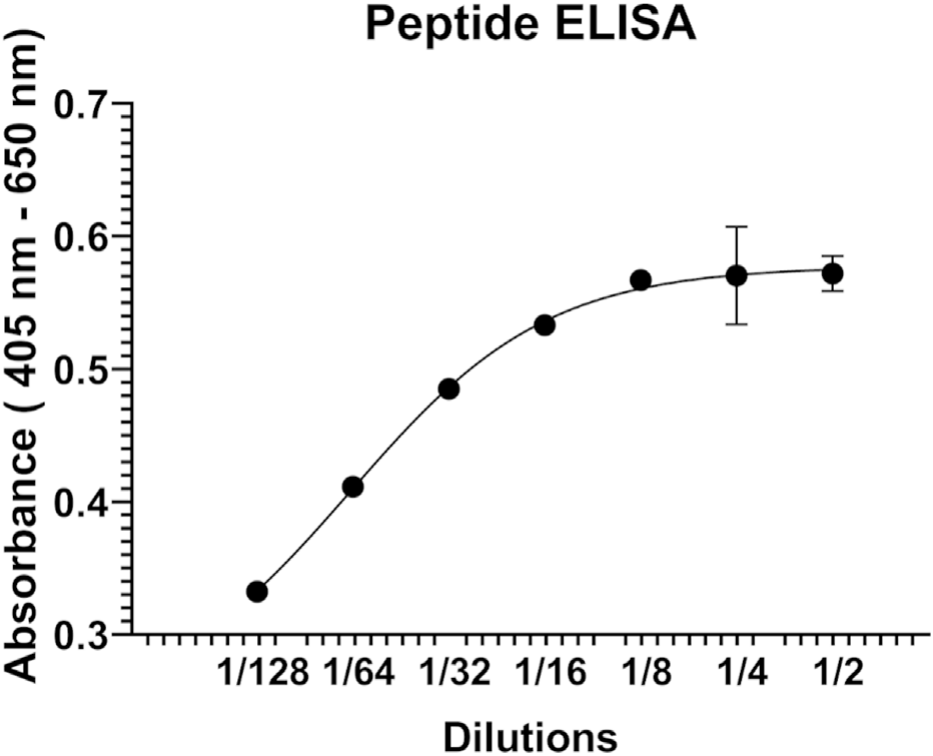
Representative graph of the peptide ELISA experiments. P1 was immobilized on the surface of a 96-well ELISA plate and was subsequently incubated with serial dilutions (2-fold) of human recombinant ALDH3A1. A rabbit ALD3A1-antibody and an anti-rabbit secondary horseradish peroxidase antibody were utilized for detecting the P1/ALDH3A1 complex through the addition of ABTS and monitoring the increase in absorbance at 405 nm-650 nm.

Next, peptide ELISA was performed to test whether the synthetic peptide P1 interacts with the hALDH3A1 protein. Specifically, P1 was coated in abundance in a 96-well plate and subsequently, hALDH3A1 was added to each well in serial dilutions. For detecting the putative peptide/ALDH3A1 interaction a rabbit anti-ALDH3A1 antibody along with a secondary horseradish peroxidase-conjugated anti-rabbit antibody were used in a sandwich ELISA. In each step, the excessive, unbound ALDH3A1 or antibody was removed through repetitive washes, while a ready-to-use ABTS solution was used as a colorimetric substrate for detecting the presence of horseradish peroxidase activity. After determining the absorbance (405 nm–650 nm) with an ELISA plate reader, an ALDH3A1 dose-dependent increase in absorbance was observed, indicating the specific binding of P1 to ALDH3A1 (Figure 2).

### 3.3 Modeling the binding of P1 to hALDH3A1

**FIGURE 3:**
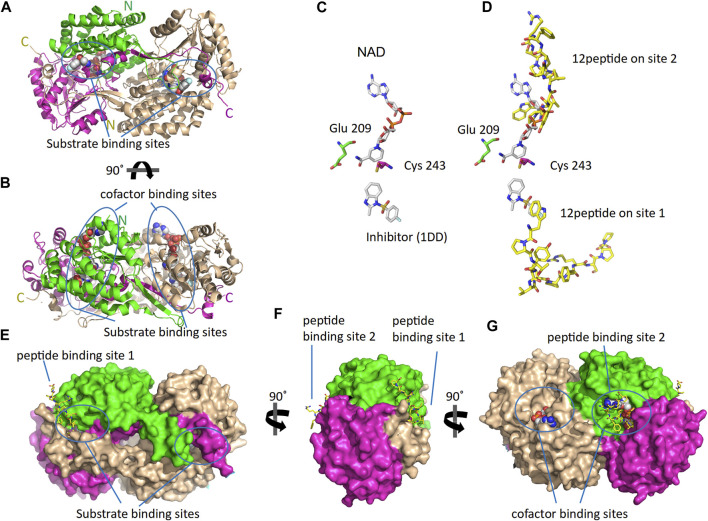
The 3D structure of ALDH3A1 dimer (PDB id 4L2O) and the predicted peptide (P1) binding sites on the protein. One monomer of the protein is wheat while the other is two-colored to indicate the catalytic (green) and the cofactor/NAD (magenta) binding domains. **(A,B)** The protein structure is shown in cartoon representation. The bound inhibitor and NAD molecules are indicated with space-filling models. Panels A and B show two different views of the molecule after a 90° rotation around the indicated axis. **(C)** The active site of one monomer. The catalytic residues Cys 243 and Glu 209 are indicated. On the substrate and cofactor binding sites are present the inhibitor 1DD (1-[(4-fluorophenyl) sulfonyl]-2-methyl-1H-benzimidazole) and the cofactor NAD (nicotinamide-adenine dinucleotide) (sticks representation). **(D)** The best HPEPDOCK solutions, for P1 binding on Sites 1 and 2, are indicated by the superposition of the peptide models (yellow sticks) on the inhibitor and cofactor molecules (gray sticks), respectively. **(E–G)** The protein structure is shown in surface representation. Docked on the protein’s surface are indicated with yellow sticks the peptide models used in panel **(D)**.

**FIGURE 4:**
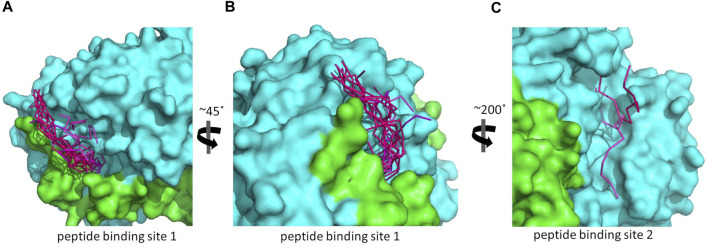
The predicted peptide (P1) conformations bound on the ALDH3A1 surface. Each protein monomer is colored differently, i.e., green and cyan. HPEPDOCK solutions are indicated by dark magenta ribbons and CABS-dock solutions by purple. **(A,B)** Site 1 (15 models). **(C)** Site 2 (2 models).

**FIGURE 5:**
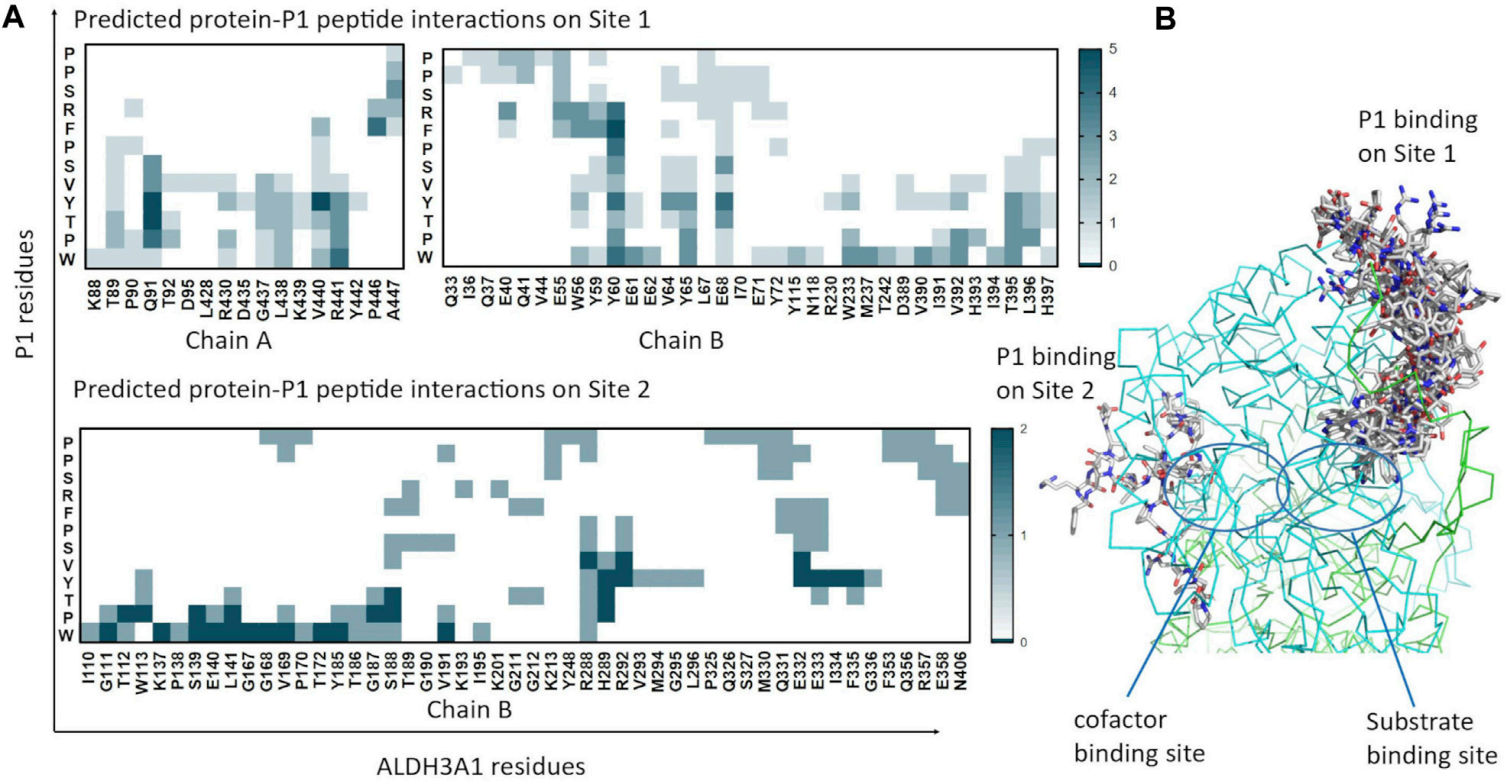
Predicted P1 conformations bound on ALDH3A1. **(A)** Heatmaps highlight the protein/peptide residues found in close proximity (less than 5.5 Å apart) at the predicted complexes. The five top solutions on Site 1 (upper panel) and the two solutions on Site 2 (lower panel) were used for the diagrams. **(B)** Superposition of the predicted P1 conformations bound on the ALDH3A1 Site 1 (15 models) and Site 2 (2 models). The protein chains are represented by green and cyan ribbons. The peptide models are shown as sticks (gray for carbon, red for oxygen, and blue for nitrogen atoms).

As reported by the results of phage display and peptide ELISA experiments, there are strong indications that the P1 has a high affinity for binding on hALDH3A1. However, the region of the protein where the peptide binds is unknown considering that there is no evidence of other hALDH3A1-oligopeptide interactions. To answer this question, we performed peptide docking on the dimeric structure of hALDH3A1 using HPEPDOCK and CABS-dock programs as it is described in the Materials and Methods section (Table 2). A total of 20 top solutions were generated by both algorithms. 15 of these solutions (75%) predict Site 1 as the peptide binding site on hALDH3A1 and 2 solutions (10%) predict Site 2 (Table 2; Figures 3–5). Visual inspection of the predicted P1 binding sites shows that they are located around either the substrate/inhibitor binding site (Site 1) or the cofactor binding site (Site 2), as it is depicted in Figures 3 and 4. Figure 5B shows that the predicted P1 models on Site 1 are well superimposed adopting quite similar conformations and common sequence direction with the first Trp to be always placed inside the substrate pocket and the rest of the peptide to wrap around the protein surface (see also Figure 4). Likewise, the two peptide models on Site 2 have common sequence directions and again Trp is almost inside the cofactor pocket. However, in one solution the peptide adopts a compact conformation and in the other an extended one (Figure 4C; Figure 5). Figures 3C and D, in particular, demonstrate the superposition of P1, when it is predicted on Site 1 and Site 2, with the inhibitor 1DD and the cofactor NAD, respectively. For this comparison, the P1 models generated from HPEPDOCK as best solutions for Sites 1 and 2 were used. The complex structure of hALDH3A1 with the 1DD inhibitor (1-[(4-fluorophenyl) sulfonyl]-2-methyl-1H-benzimidazole) and the cofactor NAD (nicotinamide-adenine dinucleotide) is experimentally determined and deposited with Protein Data Bank (PDB id 4L2O) (Parajuli et al., 2014b). The significance of the predicted solutions is also demonstrated by the numerous protein/peptide residues which are located in close proximity (less than 5.5 Å) to the generated models (Figure 5A; Table 3). It is expected that the binding of P1 on either of the predicted sites will impair the enzymatic activity and/or modify the substrate specificity.

**TABLE 2:** Summary of the ranking parameters for the top 10 models from HPEPDOCK and CABS-dock.

HPEPDOCK		CABS-dock			
Model rank	Docking score	Model rank	Cluster density	Average RMSD	Max RMSD
1	−258.923	1	53.4325	1.89023	22.9574
2	−238.125	2	42.9727	3.00191	12.0226
3	−233.652	3	30.8933	3.33406	6.7481
4	−228.486	^a^4	16.355	5.01377	21.6894
^a^5	−217.473	5	6.26563	5.72006	15.2828
6	−216.912	6	9.0856	5.94347	33.3174
7	−214.430	7	8.91125	5.16202	36.965
8	−214.403	8	6.33321	2.21057	3.74216
9	−213.480	9	3.64805	5.75649	12.6035
10	−212.724	10	2.55977	21.4863	50.4724

^a^ HPEPDOCK, model 5 and CABS-dock model 4 dock P1 at Site 2. All the other HEPDOCK models and the CABS-dock models 1–3, 5, 7, and 9 dock P1 at Site 1.

**TABLE 3:** Summary of the most prominent interacting residues between ALDH3A1 and P1 at the two predicted binding sites (PRODIGY analysis).

Site 1				Site 2	
ALDH3A1 residues (chain A)	P1 residues	ALDH3A1 residues (chain B)	P1 residues	ALDH3A1 residues (chain B)	P1 residues
Q91	P2, T3, Y4, V5, S6	E55	R9	G111	W1
V440	Y4, F8	W56	R9	T112	P2
R441	W1, P2, T3, Y4	Y60	W1, P2, Y4, S6, P7, F8, R9	W113	P2
P446	F8	E61	W1	K137	W1
A447	S10	V64	Y4	S139	W1, P2
		Y65	W1, P2, Y4	E140	W1
		E68	T3, Y4, S6	L141	W1, P2
		W233	W1	G167	W1
		M237	W1	G168	W1
		V390	W1	V169	W1
		V392	W1, P2	T172	W1
		T395	W1, P2, T3	Y185	W1
		L396	P2	G187	P2
				S188	P2, T3
				V191	W1
				R288	V5
				H289	P2, T3, Y4
				R292	Y4, V5
				E332	Y4, V5
				E333	Y4
				I334	Y4
				F335	Y4

For Site 1, the top four models generated from HPEPDOCK and the best model of CABS-dock are used. For Site 2, HPEPDOCK model five and CABS-dock model 4 are used. The table presents interactions that are present in at least 3/5 of models on Site 1 and 2/2 of models on Site 2. The complete list of interacting residues is shown in the heatmaps of Figure 5A.

**FIGURE 6:**
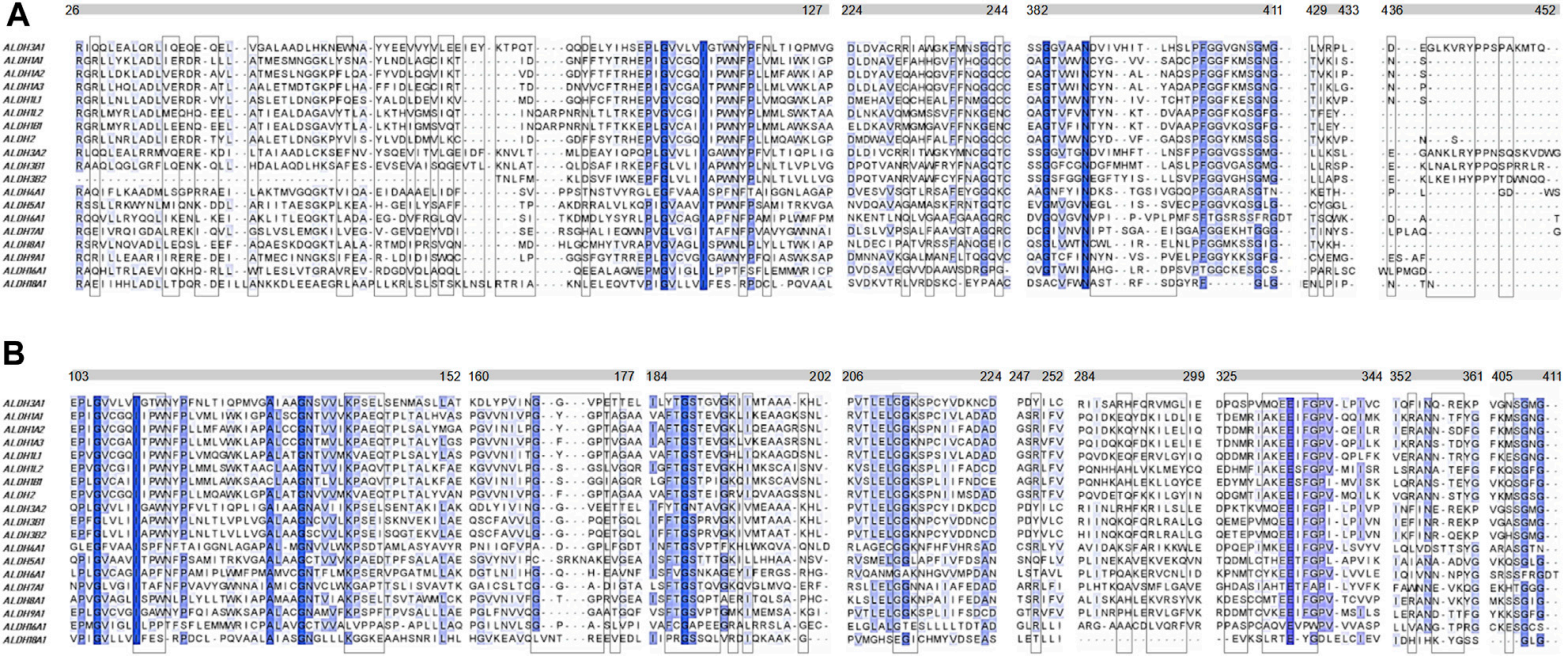
Multiple sequence alignment of the human ALDH superfamily. Selected sections from a multiple sequence alignment of the 19 human ALDH isoforms are shown. **(A)** Comparison of 5 (five) sequence segments which form the site 1 (boxed residues) i.e., the substrate binding site of the hALDHs. **(B)** Comparison of 9 (nine) sequence segments which form the site 2 (boxed residues) i.e., the co-factor binding site of the hALDHs (see also Figure 5A; Table 3). The different segments are indicated with the bars above the alignments. The numbering corresponds to the hALDH3A1 sequence. Degrees of sequence conservation are shown by different shades of blue. The darkest color indicates strict conservation within the hALDH superfamily. The lightest color corresponds to a conservation threshold of 42% sequence identity.

Another question that arises is whether the P1 is selective for hALDH3A1 or if it possibly binds to other enzymes of the hALDH superfamily as well. A multiple sequence alignment of all the hALDH isoforms (Figure 6) indicates that hALDH3A1 residues of Site 1 participating in P1 interactions are not conserved, not even among the hALDH3 subfamily (Figure 6A). In this case, P1 could be specific for hALDH3A1. In contrast, Site 2 contains amino acids that are partially conserved between the members of the superfamily (Figure 6B). If P1 binds Site 2, it could be a general interactor partner for several members of the hALDH superfamily.

### 3.4 P1 inhibits hALDH3A1 activity against benzaldehyde

**FIGURE 7:**
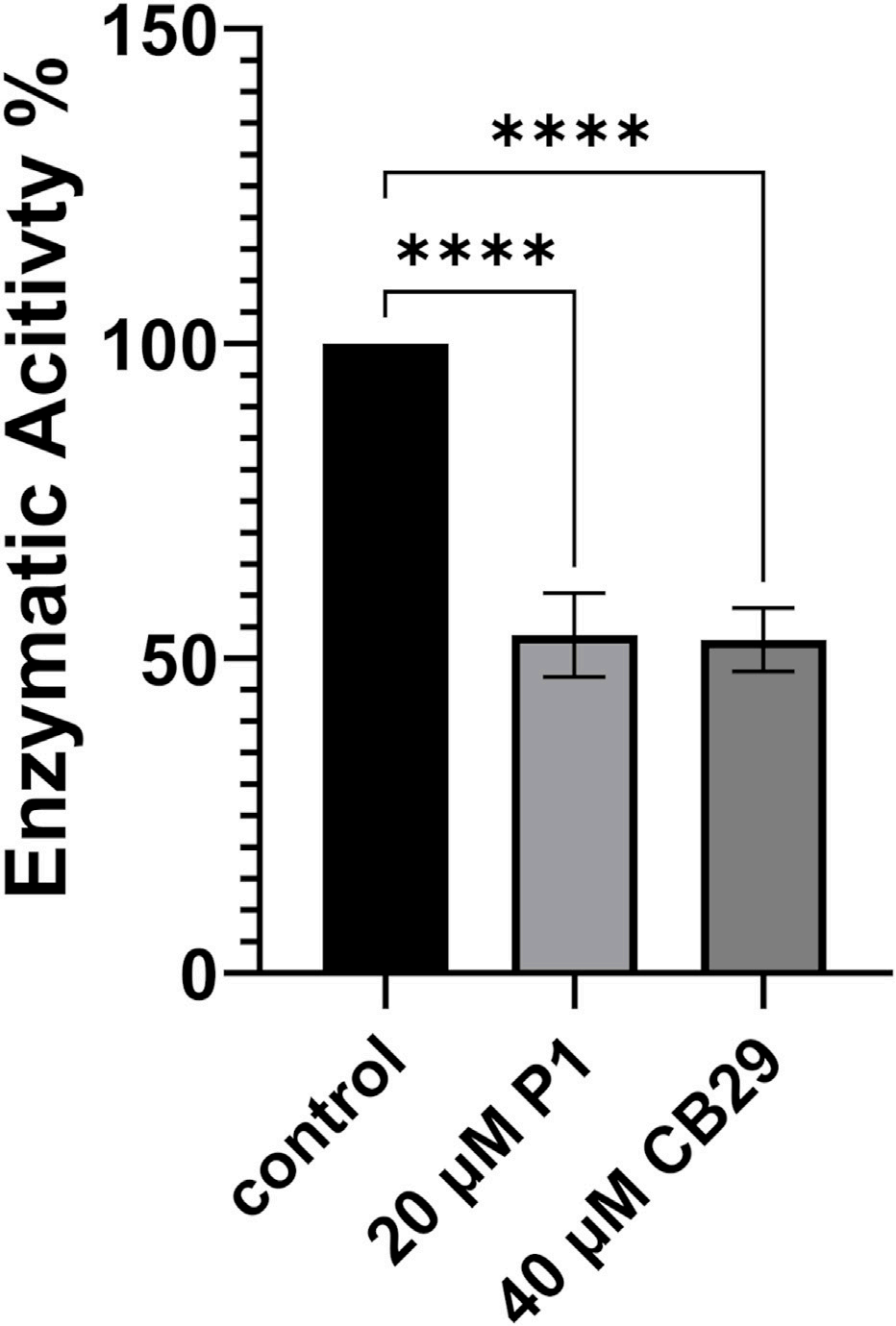
Enzymatic Activity of ALDH3A1 in the presence of P1 or CB29. P1 Inhibits the enzymatic activity of ALDH3A1 against benzaldehyde and exhibits similar effectiveness to CB29. Representative figure of at least three experiments. Results are shown as mean ± SD ****p < 0.0001 vs. control (20 nM ALDH3A1).

Benzaldehyde is a specific substrate to hALDH3A1 which oxidizes it to its corresponding benzoic acid. P1 binding either on Site 1 or 2 is expected to impair the enzymatic activity and/or to modify the substrate specificity of hALDH3A1. To further test the effect of P1 on hALDH3A1 function we assayed the ability of hALDH3A1 to metabolize benzaldehyde alone, and compared the potential inhibitory effect of P1 to the well-characterized hALDH3A1 inhibitor CB29 (Parajuli et al., 2011). Our data demonstrated that P1 at a concentration of 20 μM caused approximately a 50% reduction in hALDH3A1 activity against benzaldehyde. Similar levels of enzymatic inhibition were obtained with CB29 at a concentration of 40 μM. Overall, the results obtained with the P1 peptide appear to be comparable to the inhibition capacity of the known hALD3A1 inhibitor CB29 (Figure 7).

### 3.5 P1-based prediction of protein hALDH3A1 partners

Next, we were prompted to identify proteins that in their primary structures include a sequence identical or similar to P1. Such proteins would be potential partners of ALDH3A1 interacting through their P1-like site. First we searched the STRING database but none of the suggested protein partners includes a P1-like sequence. Next, the BLASTp algorithm was utilized to search the PDB, UniProt, RefSeq, and non-redundant databases for proteins that contain the P1 sequence. The search was first done without ‘organism’ filter and then restricted to human proteins.

The results from BLASTp showed that no protein contains the full-length sequence of P1. The search against the full database resulted in *Phytophthora boehmeriae* DNA excision repair protein ERCC-6-like 2 with nine identical, consecutive residues, i.e., TYVSPFRSP and a *Micrococcales* bacterium hypothetical protein with seven identical, consecutive residues. However, since our main interest is in partners of the human ALDH3A1 the rest of our analysis was focused on proteins resulted from the restricted to human proteins search. We noted that proteins including the highest percentage of P1 sequence had a determined 3D structure. Therefore, our analysis was focused on results against the PDB database. Table 4 summarizes the proteins that include in their sequences a peptide highly similar to P1.

**TABLE 4:** Proteins that match the P1 sequence emerged from BLASTp search against the PDB database.

Matched proteins	Alignment	Subcellular compartment	Tissue expression	E-Values
Protein kinase C-binding protein 1 (PDB id 5B73)	Query: SPFRSP Subject: SPFRTP	Nucleus, cytosol	All tissues (highest expression in brain, lung, pancreas, and placenta)	24
General transcription factor II-I (PDB id 2ED2)	Query: PFRSP Subject: PFRSP	Nucleus, cytosol	All tissues	24
Pseudopodium-enriched atypical kinase 1 (PDB id 6BHC)	Query: PFRSP Subject: PFRSP	Cytoskeleton, focal adhesions	All tissues	24
tRNA (adenine (58)-N (1))-methyltransferase catalytic subunit TRMT61A (PDB id 5CCB)	Query: SPFRS Subject: SPFRS	Nucleus	All tissues	49
Mitogen-activated protein kinase 13 (PDB id 3COI)	Query: TYVSP Subject: TYVSP	Nucleus, cytosol	Testes, pancreas, small intestine, lung, and kidney	34
Cullin-4B (PDB id 4A0C)	Query: WPTYV Subject: WPTYV	Nucleus	All tissues	2.9
Serine/threonine-protein kinase DCLK1 (PDB id 5JZN)	Query: PTYVSP Subject: PTYVAP	Plasma membrane	Ubiquitously in the brain, detectable in the heart, liver, spleen, thymus, prostate, testis, ovary, small intestine, and colon	17
Tribbles homolog 1 (PDB id 5CEK)	Query: PTYVSP Subject: PAYVSP	Nucleus, cytosol	All tissues (highest expression in skeletal muscle, thyroid gland, pancreas, peripheral blood leukocytes, and bone marrow)	34
Alpha-actinin-4 (PDB ID 6O31)	Query: TYVSPF Subject: TYVSSF	Nucleus, cytosol	All tissues	49
Glutaredoxin-3 (PDB id 2YAN)	Query: WPTY Subject: WPTY	Cytosol	All tissues (highest expression in heart, spleen, testis)	34
Transcription elongation factor A protein 1 (PDB id 5IY6)	Query: TYVSPF-RSP Subject: TYVSSFPRAP	Nucleus	All tissues	5.9

For each protein, the table includes the query (P1 sequence, upper line) and subject (matched protein sequence, bottom line) sequence alignments, the subcellular localization, the tissue expression of each protein, and the BLAST E-Values. The proteins are listed starting from those which include the largest number of consecutive identical or similar to P1 sequence, residues.

Given that the full-length P1 was not found in any protein of the database (Table 4), the possibility for each of the proteins of Table 4 to be a hALDH3A1 partner through P1-like interactions was evaluated taking into account the following criteria: 1) the number of consecutive residues which are identical or similar to the P1 sequence (see Table 4), 2) the localization of the P1-like sequence in the protein structure, i.e., on the protein’s surface or buried within the protein structure, 3) the protein’s subcellular localization and tissue expression and whether this localization is consistent with hALDH3A1’s localization. Taking into account the above criteria, we considered that a cytoplasmic or a nuclear protein with a high percentage of P1-sequence exposed on its surface has a higher potential to interact with hALDH3A1 through a P1-like interaction sequence than any extracellular or mitochondrial candidate whose P1-like sequence is buried or semi-buried in the protein interior.

Consistent with the above, three proteins in Table 4 with notably low E-values were not selected for further analysis. In particular, although Cullin 4b has the lowest E-value, it was not selected as a good candidate because the P1-like sequence, WPTYV, is semi-buried in the protein’s interior. Similarly, Transcription elongation factor A protein 1 has a low E-value but it contains a gap in its P1-like sequence, along with two more mismatches, therefore it was considered that the original P1 sequence is not well represented in this protein. Moreover, Serine/threonine-protein kinase DCLK1 was excluded from further analysis because it is a plasma membrane protein with the P1-sequence only partially exposed on its surface.

**FIGURE 8:**
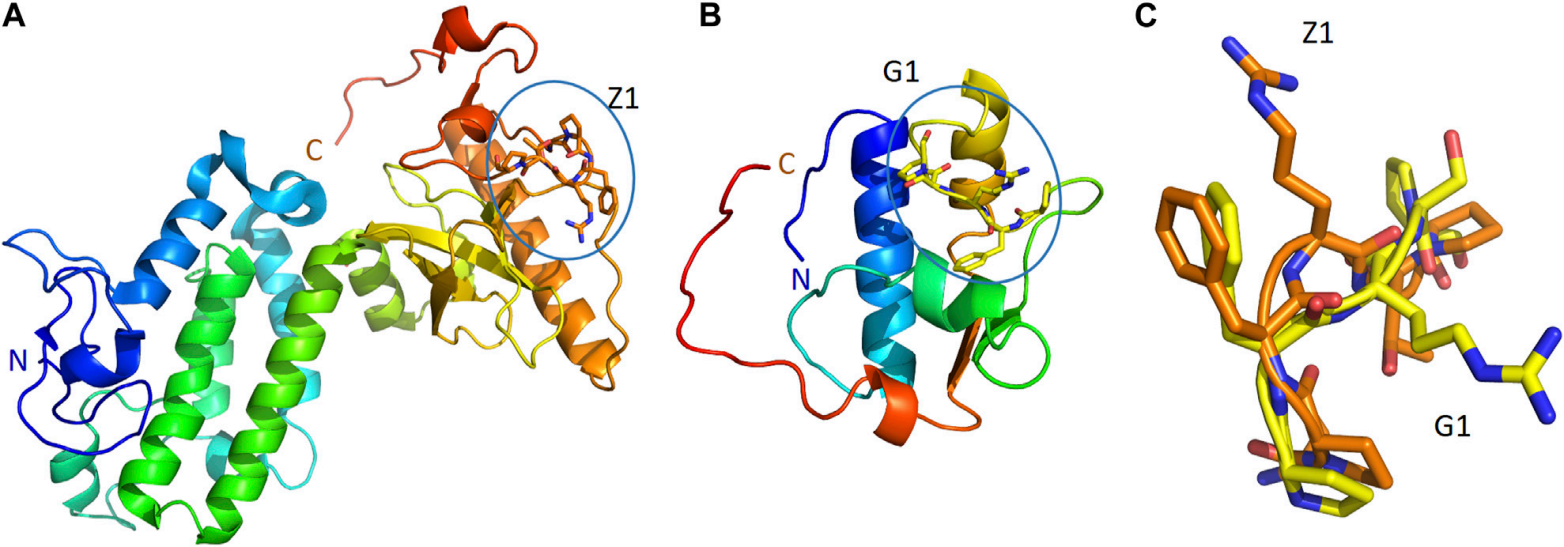
P1-like sequences, and their comparison, in two, predicted ALDH3A1 partners. The structures of PRKCBP1 (PDB id 5B73) **(A)** and GTFII-I (PDB id 2ED2) **(B)** in cartoon representation. Coloring blue to red from N-terminus to C-terminus. The conformations of Z1 (orange sticks) and G1 (yellow sticks) peptides as parts of PRKCBP1 and GTFII-I are also shown in panels **(A,B)**, respectively. **(C)** Superposition of the conformations that Z1 and G1 adopt in the structures of PRKCBP1 and GTFII-I.

On the other hand, our criteria highlighted two proteins of Table 4 as dominant candidates for hALDH3A1 interactions and they were selected for further examination (see Predicted interaction sites of PRKCBP1 and GTFII-I with hALDH3A1). These proteins are 1) The Protein kinase C-binding protein 1 (PRKCBP1, also known as ZMYND8) which is a partner protein of Protein Kinase C (PKC) and contributes to increasing its phosphorylation activity, and (ii) The General Transcription Factor II-I (GTFII-I) which is a protein that holds various roles in the regulation of gene expression. PRKCBP1 is expressed in all tissues, in the cytosol and the nucleus. Moreover, the protein contains the 6-peptide SPFRTP which is identical to the P1 SPFRSP sequence except for the minor substitution of threonine to serine at the fifth place (Figures 8A, C). Likewise, GTFII-I is a protein expressed in all tissues in nucleus and cytosol, and contains the 5-peptide PFRSP, identical to a fragment of the P1 sequence (Figures 8B, C).

### 3.6 Predicted interaction sites of PRKCBP1 and GTFII-I with hALDH3A1

We further investigated the possible interacting sites of hALDH3A1 for each of the two dominant candidates mentioned above, i.e., the Protein Kinase C binding Protein 1 (PRKCBP1) and the General Transcription Factor II-I (GTFII-I). For this purpose, we used the GalaxyTongDock_A server. The server searched the whole ALDH3A1 surface to predict the optimal sites for docking the P1-like sequence of each of the two candidate proteins. Interestingly, both proteins share an almost common P1-like sequence, namely, SPFRTP (hereafter referred to as Z1) for PRKCBP1 and PFRSP (hereafter referred to as G1) for GTFII-I (Table 4). In both proteins, the peptide is part of a long, surface-exposed random coil indicating high flexibility and an ability for participating in protein-protein interactions (Figure 8).

**FIGURE 9:**
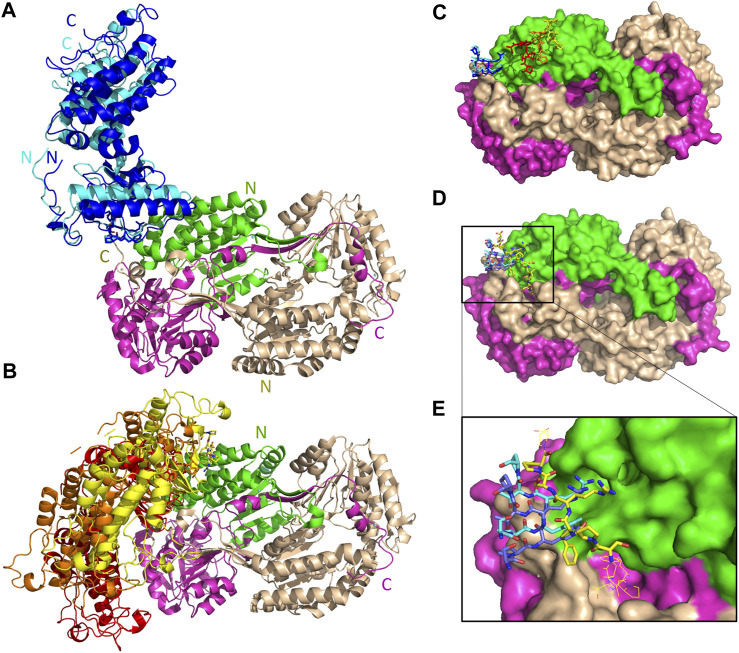
Interaction sites of the predicted ALDH3A1-PRKCBP1 complexes. ALDH3A1 coloring is as in Figure 3. The top five docking solutions are shown in panels **(A)** top 1 and 5 and **(B)** top 2–4. **(C)** The five Z1 docking conformations are shown on the ALDH3A1 surface (stick models). Two of them (blue stick models) overlap with P1 at binding Site 1 which in **(D)** is indicated by the yellow stick model. **(D)** Superposition of predicted P1 conformation bound on Site 1 (best HPEPDOCK docking solution) with two out of the five Z1 predicted conformations. **(E)** Zoom-in of **(D)**.

**FIGURE 10:**
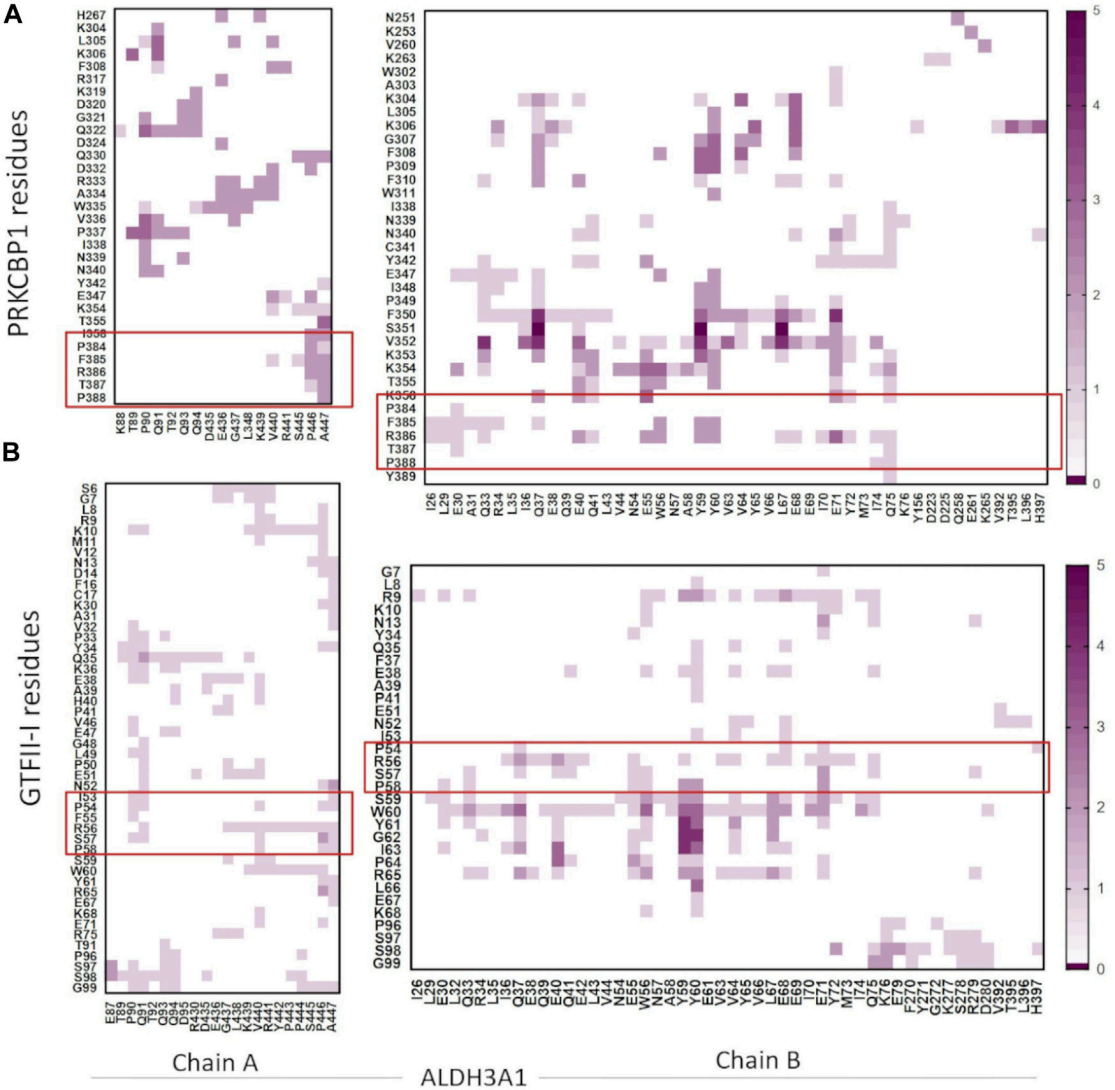
Heatmaps demonstrate the predicted protein-protein interfaces, highlighting residues found in close proximity, i.e., 5.5 Å or less apart. **(A)** ALDH3A1-PRKCBP1 and **(B)** ALDH3A1-GTFII-I predicted interfaces, respectively. The five top solutions were used for the diagrams. The red boxes indicate the Z1/G1 sequences involved in the interactions.

The five top solutions generated by GalaxyTongDock_A server, dock PRKCBP1 at the same region of hALDH3A1, close to the substrate binding site (Figure 9). PRKCBP1 is docked with two different orientations which both bring the Z1 to the rim of the substrate binding pocket and block it either fully or partially (Figures 9A, B). Notably, one of these orientations brings Z1 to a position that fully overlaps with the P1 binding Site 1 (Figures 9C–E). Figure 10A demonstrates the predicted protein-protein interfaces in all five top models. In particular, the protein-protein interactions established in the top 1 solution, which is one of those locating Z1 at the P1 binding Site 1 were further analyzed by the PDBePISA server. The analysis identified 24 interacting residues of hALDH3A1 (3 from chain A and 21 from chain B), and 26 of PRKCBP1. Eight (8) hydrogen bonds and seven (7) salt bridges are formed and stabilize PRKCBP1 on chain B of hALDH3A1. 5 out the 6 Z1 residues, namely, Pro384, Phe385, Arg386, Thr387, Pro388 participate in interactions with hALDH3A1. Especially, Arg386 may create four salt bridges with Glu40 and Glu55 and a hydrogen bond with Glu55 of hALDH3A1 chain B. A total area of 905,2 Å^2^ is buried upon hALDH3A1/PRKCBP1 complex formation. In addition, PRODIGY analysis of the model indicates a highly stable complex with a calculated binding affinity (ΔG) of −10.4 kcal mol^−1^, and a dissociation constant (K_d_) of 46 nM.

**FIGURE 11:**
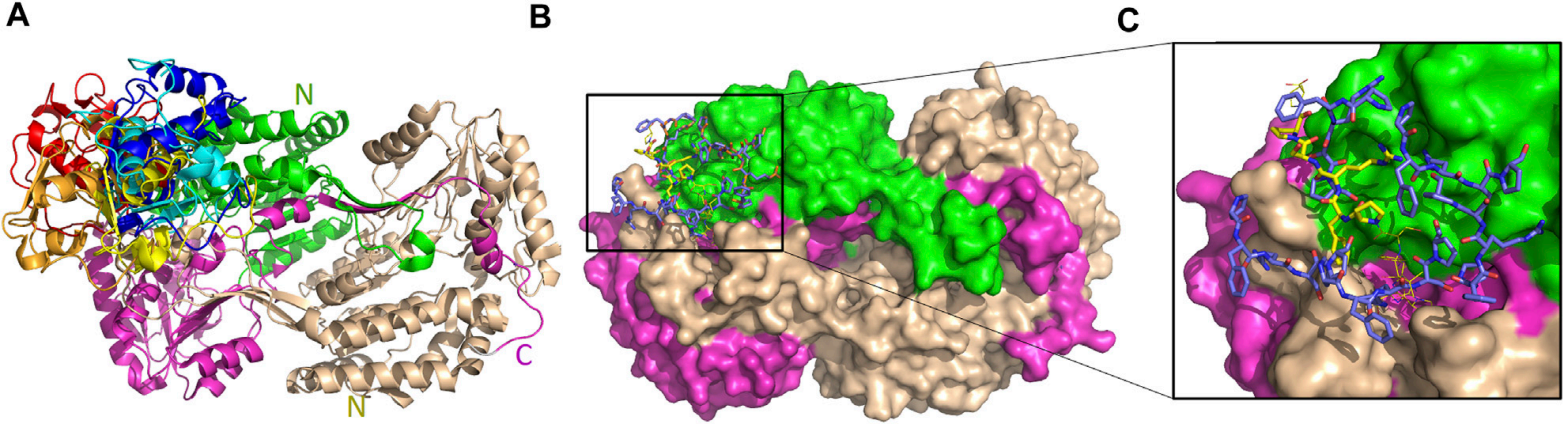
Interaction sites of the predicted ALDH3A1-GTFII-I complexes. ALDH3A1 coloring is as in Figure 3. The top five docking solutions are shown in panel **(A)**. **(B)** The top five G1 docking conformations (blue sticks) are shown on the ALDH3A1 surface. P1 binding Site 1 is also indicated by the yellow stick model which represents the best HPEPDOCK P1 predicted conformation. **(C)** Zoom-in of **(B)**.

The five top solutions generated by GalaxyTongDock_A server, dock GTFII-I around the substrate binding site of hALDH3A1, are quite similar to the predicted PRKCBP1 interaction site on hALDH3A1 (Figure 11A). The predicted G1 binding sites are scattered across the upper rim of the substrate binding pocket in close proximity to the P1 binding Site 1 as it is depicted in Figures 11B, C. Figure 10B demonstrates the predicted protein-protein interfaces in all five top models. In particular, the protein-protein interactions in the top 1 solution are further analyzed by PDBePISA. 32 residues of GTFII-I and 115 of hALDH3A1 (66 of chain B and 49 of chain A) are participating in the formation of the interface. From these residues, Pro54, Phe55, and Arg56 belong to G1. Especially, Arg56 forms a hydrogen bond and two salt bridges with Glu71 of chain B of ALDH3A1. A total area of 1151,8 Å^2^ is buried upon hALDH3A1/GTFII-I complex formation. The ΔG and the K_d_ of the model as calculated by the PRODIGY server are −11.7 kcal mol^-1^ and 5.2 nM, respectively.

## 4 Discussion

Human ALDH3A1 is an important antioxidant enzyme possessing multiple cytoprotective properties in corneal homeostasis. Additionally, it has recently emerged as a potential cancer stem cell marker in several malignancies. In this study, we used a phage peptide display library and isolated four hALDH3A1-interacting peptides, one of which (P1) appeared to bind most efficiently to hALDH3A1 and therefore was further analyzed. Peptide ELISA confirmed the ALDH3A1-peptide interactions and computational analysis predicted two potential binding sites of P1 to hALDH3A1. The most prominent of the two (Site 1) is located in the vicinity of the substrate binding site and the other (Site 2) is at the NAD binding pocket. Enzymatic studies verified that P1 has an inhibitory effect on hALDH3A1’s catalytic activity against its specific substrate benzaldehyde. Finally, we asked whether the P1 is found in a protein’s sequence. A BLASTp search identified many proteins containing parts of the P1 sequence, though none of them contains the full-length 12-residue P1. Among them, Protein kinase C-binding protein 1 and General transcription factor II-I were assessed as dominant interacting candidates and putative biological partners of hALDH3A1 taking into account their tissue expression, functional/biological role, and sub-cellular localization. Applying computational methods, the potential interaction sites of hALDH3A1 with these two proteins through the P1-like sequences were predicted and the feasibility and stability of interactions were evaluated based on the number of interacting residues and the accessible surface area buried upon proteins’ association.

Phage peptide display is a powerful tool for identifying peptide-protein interactions. It is convenient for selecting from a wide pool of random peptides and therefore, usually leads to a plethora of results (Liang et al., 2021). Phage display utilizes the properties of filamentous phages, like f1, fd, and M13 (with M13 being the most used). These phages present a minor coat protein III on their surface, so insertion of a foreign sequence in its gene results in the display of this protein/peptide, which is fused with pIII, on the surface of the phage where it can actively interact with a target protein (Ebrahimizadeh and Rajabibazl, 2014). By inserting a variety of random sequences in the gene of pIII, a fusion phage library can be produced and used for the simultaneous examination of all the proteins/peptides of the library considering their ability to interact with the desired target protein. The Ph.D.-12 Phage Display Peptide Library Kit that we used is a library constructed by approximately 2.7 × 10^9^ random 12-mer peptides fused at the pIII coat protein of the M13 phage. These peptides were screened for their ability to interact with the human recombinant 6-His tagged ALDH3A1. After 3 panning rounds, we identified four different peptides, however, we focused only on the P1 as it was present on 30 out of the 33 isolated clones. Peptide ELISA was used next to verify the P1/ALDH3A1 interaction. The assay utilized a sandwich ELISA approach in which the peptide P1 was captured on a solid surface and was performed quantitatively with ALDH3A1 in serial dilutions for accessing the binding activity to P1. The results of the peptide ELISA confirmed the interaction of ALDH3A1 with the synthetic peptide P1 in a concentration-dependent manner.

The following *in silico* analysis aimed to investigate 1) the possible P1 binding sites on hALDH3A1 and alternatively 2) the possibility of P1 being the interaction site between hALDH3A1 and other functional protein partners. Indeed, our computational analysis confirmed that P1 has a binding affinity for hALDH3A1 and predicted two possible protein sites, namely, Site 1 and Site 2, for specific peptide binding. Site 1 is extended around the substrate binding site and it is the most prominent interaction site as it is indicated by the vast majority of the generated models. On the other hand, Site 2 overlaps with the NAD(P)^+^ binding region of hALDH3A1. Peptide binding on either of the two sites is expected to cause hALDH3A1 inactivation or at least alteration of the enzyme’s substrate specificity. Indeed, enzymatic studies showed that in the presence of P1, hALDH3A1 exhibits reduced activity against its specific substrate benzaldehyde. The reduction is comparable with the inhibition caused by the CB29 molecule, which is a synthetic hALDH3A1 inhibitor developed by Parajuli et al. (2014b). Further computational analysis of the possible sites showed that Site 1 is specific for hALDH3A1 while Site 2 displays at least partial conservation among different isoforms of the human aldehyde dehydrogenase superfamily. This observation implies that P1 could prospectively act as a specific hALDH3A1 modifier if binds Site 1 or as a general hALDH family modifier if binds Site 2. Our methodology is in line with previously published studies utilizing peptide phage display to identify specific cancer and/or protein-targeting peptides (Shi et al., 2015; Li et al., 2017; Zhao et al., 2021). Nevertheless, to the extent of our knowledge, it is the first time that a peptide has been proposed as an ALDH modifier. In general, therapeutic peptides are gaining increasing interest over the last few years due to their efficacy, safety, low toxicity, and high selectivity (Fosgerau and Hoffmann, 2015).

The second goal of our computational analysis was to use the P1 sequence as a lead in proteins that may interact with hALDH3A1 through a P1-like sequence. Thus, we performed BLASTp to screen PDB, UniProt, RefSeq, and non-redundant databases for human proteins containing the P1 sequence. Considering that none of the proteins contains in its sequence a full-length P1 peptide, we extracted a list of proteins containing at least a P1 sequence fragment. To this end, we listed 11 proteins (Table 4) including transcription factors (General transcription factor II-I, Transcription elongation factor A protein 1), kinases or kinase-related proteins (Protein kinase C-binding protein 1, Pseudopodium-enriched atypical kinase 1, Mitogen-activated protein kinase 13, Serine/threonine protein kinase DCLK1, Tribbles homolog 1) and enzymes (tRNA (adenine (58)-N (1))-methyltransferase catalytic subunit TRMT61A, Glutaredoxin-3). Next, we used the following criteria to select from the above list the most probable P1-based functional partners of hALDH3A1: 1) the maximum number of consecutive identical or physicochemically similar residues, 2) the localization of the P1-like sequence at the surface of the protein, 3) the tissue and sub-cellular expression pattern in comparison to hALDH3A1 and 4) the biological functions of the proteins. Two proteins were highlighted as the most promising candidates on the list, namely, the Protein Kinase C-binding protein 1 (PRKCBP1) and General Transcription factor II-I (GTFII-I).

PRKCBP1 is a member of the receptor for the activated C-kinase (RACK) family. The RACK proteins anchor activated PKC to increase its phosphorylation and prolong its activation period (Fossey et al., 2000). PRKCBP1 is associated with the DNA-damage response (DDR) by repressing transcription and promoting homologous recombination to repair DNA double-strand breaks (DSBs) (Gong et al., 2015). PRKCBP1 also appears to be highly involved in cancer as an epigenetic regulator of tumor suppression (Li et al., 2016). In breast cancer cells, PRKCBP1 is a hypoxia-induced epigenetic reader that interacts with hypoxia-inducible factors (HIFs) 1α and 2α, to amplify HIF-mediated activation of oncogenes, subsequently increasing breast cancer progression and metastasis (Chen et al., 2018). Its involvement in many different pathways, along with its localization in both the cytoplasm and the nucleus, led us to single out this protein as an important candidate for interactions with ALDH3A1. This speculation was also supported by the fact that this particular scaffold protein creates many interactions, either directly or as part of complexes, through its various domains, such as SH2 domains (Src and Fyn), plextrin homology (PH) domains (dynamin and p120GAP) and C2 domains (PKCs) (Adams et al., 2011). Moreover, it is known that other family members of the ALDH superfamily are phosphorylated by PKCs, for example, the ALDH2 by the εPKC (Chen et al., 2008). Interestingly, using Motif Scan by MyHits we found that the amino acid sequence of hALDH3A1 contains four possible phosphorylation sites which can be recognized by PKCs. These sites, which match the PROSITE registered PKC recognition motif S/T—x –R/K (PS00005), are located at the positions 17–19 (SGR), 267–269 (SLK), 287–289 (SAR) and 423–425 (SHR) of hALDH3A1. Three of the above sites are located at the surface of the protein, while the last one (SHR) is found in a pocket at the dimers interface.

General transcription factor II-I (GTFII-I) is a multifunctional, ubiquitously expressed protein with both cytoplasmic and nuclear functions (Roy, 2007; Roy, 2017). In the cytoplasm, GTFII-I plays a key role in calcium signaling by reducing Ca^2+^ influx (Caraveo et al., 2006). In the nucleus, it can act either as a transcriptional activator or repressor. Upon growth factor and mitogenic stimuli, GTF-II associates with the c-fos promoter and positively regulates its transcription, *via* Ras/Erk and Rho signaling pathways (Cheriyath et al., 2002; Roy, 2017). Additionally, GTFII-I is involved in the transcriptional activation of the cyclin D1 gene that results in accelerated entry to and exit from the S phase (Desgranges et al., 2005), while it seems to be downstream of TGF-β signaling (Ku et al., 2005) and ER stress response pathways (Parker et al., 2001). Finally, GTFII-I is associated with breast cancer, as it is found to interact with estrogen receptor *a* (ERα) and represses the transcription of estrogen-responsive genes. Therefore, by negatively regulating these pro-proliferative genes, GTFII-I may have a crucial role in inhibiting tumor growth (Ogura et al., 2006). Moreover, it has been demonstrated that GTFII-I interacts with BRCA1, a tumor suppressor gene product mutated in breast and ovarian cancers (Tanikawa et al., 2011). According to that study, BRCA1 acts as a transcriptional co-factor for GTFII-I, for the expression of target genes involved in DNA damage checkpoint pathways, such as SIRT1 and tumor suppressor p53. In addition, GTFII-I and BRCA1 nuclear co-localization was reported after γ-irradiation-induced DNA damage. As a result, GTFII-I might be involved in the activation of tumor suppressor genes resulting in the attenuation of cellular proliferation (Tanikawa et al., 2011).

Considering the common association of hALDH3A1 and the selected proteins with cancer, DNA damage response, and cell cycle regulation, we subsequently analyzed *in silico* the putative hALDH3A1:PRKCBP1 and hALDH3A1:GTFII-I complexes. Our results indicate that both proteins may bind hALDH3A1 through the same interacting site which is also similar to Site 1 of P1 binding on hALDH3A1. The formation of both complexes is based on numerous interactions with a variety of residues several of which belong to the P1 sequence. The stability of the putative complexes is also verified by an extensive surface area buried upon protein-protein interaction.

In conclusion, the findings presented here identify a novel peptide with potential biomedical applications and further suggests a list of protein candidates be explored as possible hALDH3A1-interacting partners in future studies. Furthermore, the study provides clues to signal transduction pathways in which hALDH3A1 may be involved, which warrants further investigation. At the moment, our group works on the detailed characterization of the proposed P1-hALDH3A1 interactions. Our future studies will biochemically, enzymatically, and structurally characterize the binding efficacy, binding sites, and the functional consequences of P1-hALDH3A1 interactions as well as the specificity of P1 against other members of the human ALDH family.

## Data Availability

The original contributions presented in the study are included in the article/Supplementary Material, further inquiries can be directed to the corresponding authors.
