# Supplementary material for: Identification of a peptide ligand for human ALDH3A1 through peptide phage display: Prediction and characterization of protein interaction sites and inhibition of ALDH3A1 enzymatic activity
Source: Front Mol Biosci. 2023 Mar 20;10:1161111. doi: 10.3389/fmolb.2023.1161111 (PMC10067601; doi:10.3389/fmolb.2023.1161111)
Supplement: Supplementary file 1 [file DataSheet1.ZIP › Raw Data/Raw data computational explanation .docx]

Files named Hmodel_1-10 and model_1-10 contain the peptide docking prediction at the surface of ALDH3A1 with the HPEPDOCK and CABS-dock respectively. By comparing the docking position of the peptide at the models produced by both programs, we choose the two most prominent docking positions (site 1 και site 2). For figure 3 D-G that presents sites 1 & 2 models Hmodel_1 and Hmodel_5 were respectively used. Specifically, models Hmodel_1-4, hmodel_6-10, model_1-3 and model_5,7,9 docked the peptide at site 1 (Figure 4a-b), while models hmodel_5 and model_4 docked the peptide at site 2 (Figure 4c). For figure 5 and table 3 the models hmodel_1-4 and model_1 were used for site 1, and models hmodel_5, model_4 for site 2. For figure 9 models Pmodel_1-5 were used (ALDH3A1-PRKCBP1). For figure 11 Gmodel_1-5 were utilized (ALDH3A1- GTFIIi). The heatmaps in figure 10 were generated based on all of the above models (pmodel_1-5 and gmodel_1-5).
